# Analysis of Early Biomarkers Associated With Noise-Induced Hearing Loss Among Shipyard Workers

**DOI:** 10.1001/jamanetworkopen.2021.24100

**Published:** 2021-09-03

**Authors:** Zhuang Jiang, Jiping Wang, Yanmei Feng, Daoyuan Sun, Xunmiao Zhang, Haibo Shi, Jian Wang, Richard Salvi, Hui Wang, Shankai Yin

**Affiliations:** 1 Department of Otolaryngology–Head and Neck Surgery, Shanghai Jiao Tong University Affiliated Sixth People’s Hospital, Shanghai, China; 2 Otolaryngology Institute of Shanghai Jiao Tong University, Shanghai, China; 3 Shanghai Key Laboratory of Sleep Disordered Breathing, Shanghai, China; 4 Department of Occupational Disease, Shanghai Pulmonary Hospital, Tongji University School of Medicine, Shanghai, China; 5 School of Communication Science and Disorders, Dalhousie University, Halifax, Nova Scotia, Canada; 6 Center for Hearing and Deafness, University at Buffalo, Buffalo, New York

## Abstract

**Importance:**

It is important to determine what frequencies and auditory perceptual measures are the most sensitive early indicators of noise-induced hearing impairment.

**Objectives:**

To examine whether hearing loss among shipyard workers increases more rapidly at extended high frequencies than at clinical frequencies and whether subtle auditory processing deficits are present in those with extensive noise exposure but little or no hearing loss.

**Design, Setting, and Participants:**

This cross-sectional study collected audiometric data (0.25-16 kHz), survey questionnaires, and noise exposure levels from 7890 shipyard workers in a Shanghai shipyard from 2015 to 2019. Worsening hearing loss was evaluated in the group with hearing loss. Speech processing and temporal processing were evaluated in 610 participants with noise exposure and clinically normal hearing to identify early biomarkers of noise-induced hearing impairment. Data analysis was conducted from November to December 2020.

**Main Outcomes and Measures:**

Linear regression was performed to model the increase in hearing loss as function of cumulative noise exposure and compared with a group who were monitored longitudinally for 4 years. Auditory processing tests included speech-in-noise tests, competing sentence tests, dichotic listening tests, and gap detection threshold tests and were compared with a control group without history of noise exposure.

**Results:**

Of the 5539 participants (median [interquartile range (IQR)] age, 41.0 [34.0-47.0] years; 3861 [86.6%] men) included in the cross-sectional analysis, 4459 (80.5%) were hearing loss positive and 1080 (19.5%) were hearing loss negative. In younger participants (ie, ≤40 years), the maximum rate of increase in hearing loss was 0.40 (95% CI, 0.39-0.42) dB per A-weighted dB–year (dB/dBA-year) at 12.5 kHz, higher than the growth rates of 0.36 (95% CI, 0.35-0.36) dB/dBA-year at 4 kHz, 0.32 (95% CI, 0.31-0.33) dB/dBA-year at 10 kHz, 0.31 (95% CI, 0.30-0.31) dB/dBA-year at 6 kHz, 0.27 (95% CI, 0.26-0.27) dB/dBA-year at 3 kHz, and 0.27 (95% CI, 0.27-0.28) dB/dBA-year at 8 kHz. In the 4-year longitudinal analysis of hearing loss among 403 participants, the mean (SD) annual deterioration in hearing was 2.70 (2.98) dB/y at 12.5 kHz, almost twice as that observed at lower frequencies (eg, at 3kHz: 1.18 [2.15] dB/y). The auditory processing scores of participants with clinically normal hearing and a history of noise exposure were significantly lower than those of control participants (eg, median [IQR] score on speech-in-noise test, noise-exposed group 1 vs control group: 0.63 [0.55-0.66] vs 0.78 [0.76-0.80]; *P* < .001).

**Conclusions and Relevance:**

These findings suggest that the increase in hearing loss among shipyard workers was more rapid at 12.5 kHz than at other frequencies; workers with clinically normal hearing but high cumulative noise exposure are likely to exhibit deficits in speech and temporal processing.

## Key Points

**Question:**

Is the increase of hearing loss among shipyard workers more rapid at 12.5 kHz than at lower frequencies, and do any auditory processing deficits occur before there is significant hearing loss?

**Findings:**

In this cross-sectional study of 7890 shipyard workers, hearing loss at 12.5 kHz was more rapid than at lower frequencies. Greater cumulative noise exposure was associated with auditory processing deficits among those with near-normal hearing.

**Meaning:**

These findings suggest that a 12.5-kHz threshold and auditory processing disorders could serve as early indicators of noise-induced hearing impairment.

## Teaser

This cross-sectional study examines whether hearing loss among shipyard workers increases more rapidly at extended higher frequencies and whether subtle auditory processing deficits are present in those with extensive noise exposure but little or no hearing loss.

## Introduction

Prolonged and/or intense noise exposure contributes to numerous health problems, in particular noise-induced hearing loss (NIHL) that damages the cochlea.^1,2^ Noise-induced damage not only leads to hearing loss but can make it difficult to understand speech.^3,4,5^ Although many countries have established health and safety regulations,^6^ early detection of NIHL is of great importance for prevention.

The most common biomarker used to identify NIHL is the shape of the audiogram.^7,8,9^ Historically, the so-called 4-kHz noise notch has been considered a hallmark of NIHL and is used to distinguish it from other types of sensorineural hearing losses associated with aging, ototoxic effects, and genetic factors.^7,8,10,11,12^ On the other hand, it has been suggested that notches can occur in the absence of a positive noise history.^8^ For the purpose of early detection and diagnosis of NIHL, extended high frequency (EHF) audiometry has gradually become a tool for routine clinical evaluation. Advocates point out the advantage of EHF in early identification of hearing loss due to ototoxic drugs and noise exposure.^13,14,15,16^ However, a systematic review failed to arrive at a robust conclusion regarding the advantage of EHF vs conventional audiometry.^15^ Most of the conclusions have been derived from a pool of participants with unclear durations and/or doses of noise exposure.^16,17^ In addition, many studies lacked longitudinal measurement of hearing. Therefore, the utility of EHF audiometry to test for early onset NIHL remains an open question.

Traditionally, NIHL was defined by the permanent threshold shifts (PTS), while noise exposures that do not cause PTS were considered relatively safe. However, this idea has been challenged by data from animals in which significant noise-induced damage to the synapses between inner hair cells and spiral ganglion neurons occurred without PTS.^18,19^ This subclinical hearing damage, called hidden hearing loss, is not easily detected by routine hearing tests focused on thresholds, but it could contribute to temporal processing deficits or difficulty detecting or interpreting sounds in background noise.^20,21,22^ Some of these functional deficits are believed to be due to noise-induced synaptic damage.^18,23^ Several studies have tried to verify hidden hearing loss in human participants with mixed results.^24,25,26,27^ One problem with many studies is the lack of documented history of noise exposure, heterogeneity of participants, and a limited sample size from which to draw firm conclusions.^28,29,30,31,32^ With these limitations in mind, this study aimed to assess the growth of hearing loss in the clinical and EHF ranges and to identify auditory processing disorders (APDs) among workers with noise exposure to identify early biomarkers of noise-induced hearing impairment.

## Methods

### Study Design and Subjects

Cross-sectional physical examination data were obtained from 7890 sanding, welding, metal, and cutting workers between June 2015 and June 2019. A questionnaire was filled out by each participant with demographic features; noise exposure history; type of work; smoking and alcohol drinking habits; history of major diseases, including genetic and drug-related hearing loss; and the use of hearing protection devices. This study was approved by the institutional ethics review board of the Shanghai Sixth People’s Hospital, affiliated with Shanghai Jiao Tong University, and was registered in the Chinese Clinical Trial Registry (ChiCTR-RPC-17012580). Potential consequences and benefits were explained, and written informed consent was obtained from each participant before this study. This study adheres to the Strengthening the Reporting of Observational Studies in Epidemiology (STROBE) reporting guideline.

**Figure 1.**
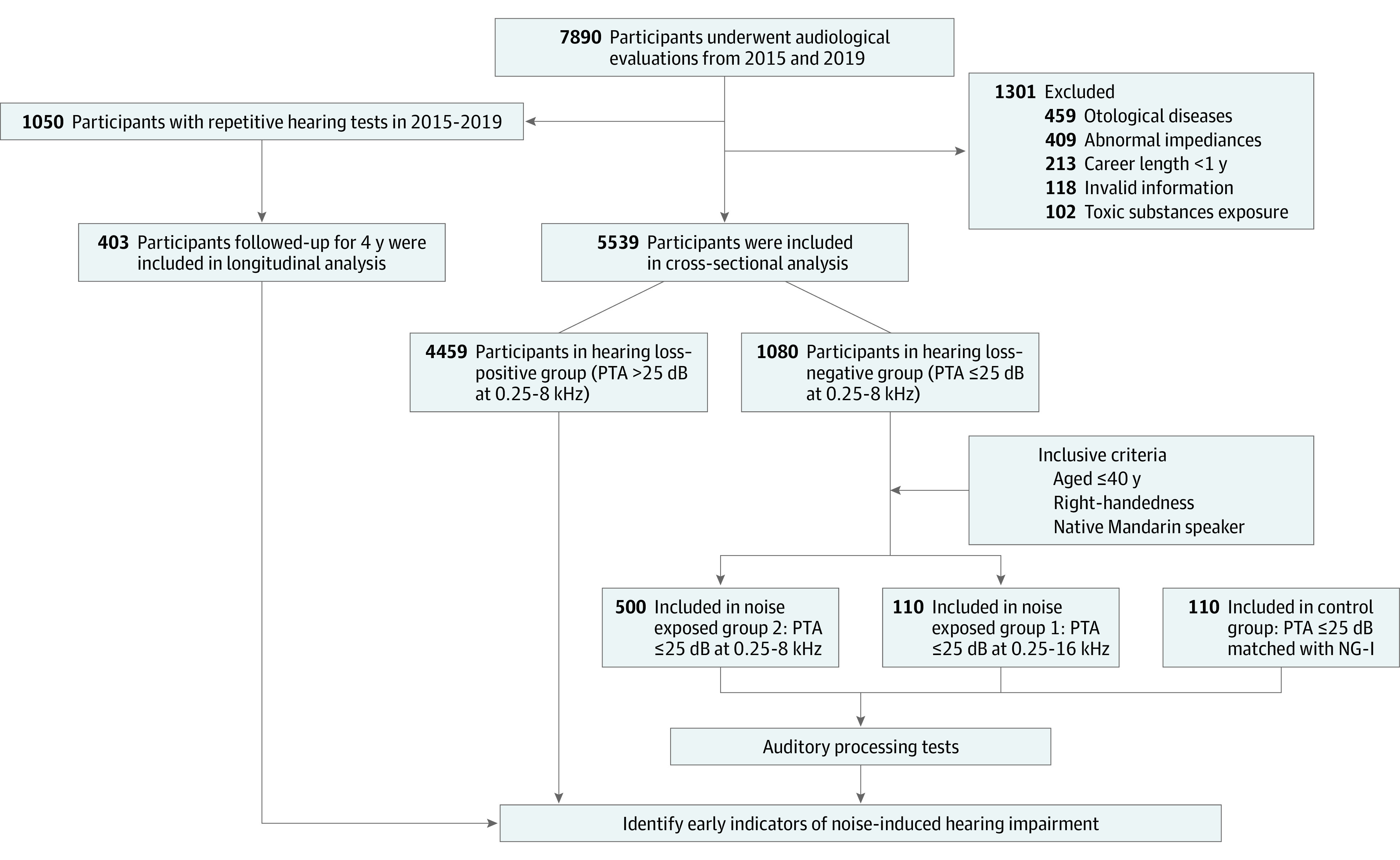
Study Flowchart PTA indicates pure-tone audiometry.

Figure 1 outlines the procedures and criteria for participant inclusion and exclusion. A total of 5539 individuals were included in the cross-sectional analysis. Among them, 4459 participants had varying degrees of hearing loss (ie, pure-tone audiometry [PTA] >25 dB hearing level at any frequency from 0.25 to 8 kHz) and were classified as hearing loss positive. The remaining 1080 participants with clinical normal hearing (ie, PTA ≤25 dB from 0.25 to 8 kHz) were classified as hearing loss negative. More extensive auditory processing tests were carried out on a subgroup of 610 participants from the hearing loss–negative group according to the following criteria: younger than 40 years, right handed, and native Mandarin speaker. Among them, 110 participants had hearing level thresholds of 25 dB in both the clinical audiometric range (ie, 0.25-8 kHz) and the EHF range (10, 12.5, and 16 kHz); this group was considered noise-exposed group 1 (NG 1). The remaining 500 participants with PTA of 25 dB or less from 0.25 to 8 kHz but PTA of greater than 25 dB at EHFs were considered noise-exposed group 2 (NG 2). Another 110 participants with normal hearing and without a history of occupational noise exposure served as the control group; these individuals were matched with those in the NG 1 group in terms of age, sex, and years of education. Furthermore, the longitudinal analysis was restricted to 403 participants younger than 40 years old at the time of the first test who were followed up for 4 years (from 2015 to 2019) to calculate a hearing threshold difference statistic at each frequency.

### Noise Exposure Estimation

Industrial noise was measured with an ASV5910-R digital recorder (Aihua Instruments) across the work areas of different jobs according to the national standard of China.^33^ Sound levels were measured using A-weighted dB (dBA). The long-term equivalent (leq) noise level was adopted as the primary exposure metric and measured 3 times at each spot. The mean value of the leq in each spot was transformed into an 8-hour continuous equivalent sound pressure level (leq-8h). Cumulative noise exposure (CNE, measured in dBA-years) was calculated using the leq-8h during the years of on-duty time, as follows: CNE = leq-8h + 10 × log(*T*), where the *T* is the career length in years.

### Audiological Evaluation

Audiologic evaluations were performed on 7890 workers on site by qualified medical assistants in soundproof chambers. The tests were performed at least 12 hours after the participant’s last shift in the noise-exposed job. Otoscopic inspection and tympanometry were performed on each participant to establish the normal function of their external and middle ears. Tympanograms were measured using a TympStar tympanometer (Grason-Stadler). The passing criterion was a type A tympanogram (peak between −100 and 100 daPa). PTA was measured separately for each ear using a Type 1066 manual audiometer (Natus Hearing & Balance) with Sennheiser HDA-300 headphones for the clinical and EHF audiometric ranges. All thresholds were calculated in dB hearing level, and audiometers were calibrated annually according to the ISO 389-5-2006 standard. If a participant did not respond to at the maximum output of the audiometer at the EHFs (90, 80, and 60 dB hearing level for 10, 12.5, and 16 kHz), the data were removed to eliminate saturation effects.

### Auditory Processing Tests

A battery of 5 auditory processing tests was performed in 2 soundproof rooms using an audiometer equipped with TDH-39 headphones; stimuli were presented at 40 dB sensation level. The speech-in-quiet test (SIQT), speech-in-noise test (SINT), and competing sentences test (CST) were assessed using the Mandarin version of the hearing-in-noise test that was developed with MATLAB version R2012a software.^34^ The dichotic listening test (DLT) was obtained from both the left ear and right ear. The gap detection threshold (GDT) was measured using a 3-interval forced-choice procedure designed with MATLAB software. The procedures used for these measurements have been described in previous studies.^35,36^ Speech recognition and DLT were scored as the percentage of correct responses. The GDT threshold (in milliseconds) was calculated from the mean value of the last 8 reversals.

### Statistical Analysis

The baseline characteristics of participants were summarized as number and percentage for categorical variables and mean and SD for most continuous variables. Median and interquartile range (IQR) were used for skewed continuous variables. Differences in demographic characteristics and results of auditory processing measurements were analyzed by the nonparametric Kruskal-Wallis test and post hoc pairwise comparison with Bonferroni correction if the data were not normally distributed. Categorical data were compared using Pearson χ^2^ test. A linear regression line with a forced intercept of 0 was fit to the data to determine the rate of hearing loss increase (slope) from 3 to 12.5 kHz, which was compared using the Z test in a younger group (ie, ≤40 years) and older group (ie, >40 years). Repeated measures analysis of variance with 2 factors (year and frequency) were used to compare the difference between hearing thresholds from 2015 to 2019. A 2-tailed *P* < .05 was considered statistically significant. Data analyses were performed using R version 4.02 (R Project for Statistical Computing) and Prism version 8.4 (GraphPad Software).

## Results

### Characteristics of Participants

The Table presents an overview of the demographic and hearing characteristics of the participants. Of the 5539 participants, 4459 (80.5%) had mild or greater hearing loss, the median (IQR) age was 41.0 (34.0-47.0) years, and 3861 (86.6%) were men. The median (IQR) career length was 7.5 (4.2-10.7) years, and the median (IQR) CNE was 92.9 (89.1-97.7) dBA-years. There were 1705 individuals (38.2%) who currently smoked, and 1685 (37.8%) who currently drank. In the hearing loss–negative group, the participants in the 3 groups (ie, NG 1, NG 2, and the control group) were fairly well matched except for PTAs. The median (IQR) PTAs at 0.25 to 2 kHz in NG 1 (9.7 [6.7-11.9] dB) and NG 2 (12.5 [9.4-15.0] dB) groups were significantly different from those in the control group (10.6 [8.8-12.5] dB; NG 1 vs control group: H = −2.247; *P* = .02; NG 2 vs control group: H = −4.675; *P* < .001), while there was no statistical difference between NG 1 and NG 2. The median (IQR) PTAs at 3 to 6 kHz and 10 to 16 kHz did not differ between the NG 1 group (3-6 kHz: 8.3 [6.7-11.7] dB; 10-16 kHz: 10.4 [6.7-13.3] dB) and the control group CG (3-6 kHz: 10.0 [7.5-12.5] dB; H = −0.524; *P* = .60; 10-16 kHz: 11.3 [8.3-16.7] dB; H = −0.130; *P* = .90), while there was a significant difference between the NG 2 group (3-6 kHz: 11.7 [8.3-15.0] dB; 10-16 kHz: 25.8 [19.2-33.3] dB) and the control group (3-6 kHz: H = −5.192; *P* < .001; 10-16 kHz: H = −15.964; *P* < .001). The mean (SD) age in the 2015 of the 403 workers in the longitudinal study was 31.7 (3.2) years, and the mean (SD) career length was 5.7 (1.7) years.

**Table.** Demographic Characteristics of the Sample

Variable	Participants, No. (%)						*P* value^a^
	Hearing loss–positive group			Hearing loss–negative group		Control group (n = 110)	
	Age ≤40 y (n = 2076)	Age >40 y (n = 2383)	Overall (n = 4459)	NG 1 (n = 110)	NG 2 (n = 500)		
Age, median (IQR), y	28.0 (25.0-29.0)	36.0 (33.0-38.0)	41.0 (34.0-47.0)	30.0 (27.0-32.0)	31.0 (28.0-34.0)	32.0 (26.0-36.0)	.06
Sex							
Male	1834 (88.4)	2027 (85.1)	3861 (86.6)	99 (90.0)	441 (88.2)	99 (90.0)	.78
Female	242 (11.6)	356 (14.9)	591 (13.4)	11 (10.0)	59 (11.8)	11 (10.0)	
Career length, median (IQR), y	4.4 (2.0-7.1)	7.0 (4.0-10.0)	7.5 (4.2-10.7)	6.0 (3.0-9.0)	6.0 (3.0-10.0)	NA	.38
CNE, median (IQR), dBA-year	90.9 (87.5-93.7)	92.2 (89.2-95.5)	92.9 (89.1-97.7)	91.4 (89.3-93.0)	91.5 (89.2-94.4)	NA	.24
Education level, median (IQR), y	9.0 (9.0-12.0)	9.0 (9.0-9.0)	9.0 (9.0-9.0)	12.0 (12.0-12.0)	12.0 (9.0-12.0)	12.0 (9.0-15.0)	.15
Smoking							
Currently	865 (41.7)	840 (35.2)	1705 (38.2)	23 (20.9)	89 (17.8)	19 (17.3)	.72
Never	1211 (58.3)	1543 (64.8)	2754 (61.8)	87 (79.1)	411 (82.2)	91 (82.7)	
Drinking							
Currently	879 (42.3)	806 (33.8)	1685 (37.8)	11 (10.0)	72 (14.4)	13 (11.8)	.41
Never	1197 (57.7)	1577 (66.2)	2774 (62.2)	99 (90.0)	428 (85.6)	97 (88.2)	
PTA, median (IQR), dB							
At 0.5-2 kHz	15.6 (12.5-18.8)	16.3 (13.1-20.0)	18.8 (15.0-23.8)	9.7 (6.7-11.9)	12.5 (9.4-15.0)	10.6 (8.8-12.5)	<.001
At 3-6 kHz	17.5 (11.7-25.8)	23.3 (15.8-35.8)	32.5 (22.5-47.5)	8.3 (6.7-11.7)	11.7 (8.3-15.0)	10.0 (7.5-12.5)	<.001
At 10-16 kHz	22.5 (12.5-35.6)	32.5 (20.0-48.8)	45.0 (34.2-56.7)	10.4 (6.7-13.3)	25.8 (19.2-33.3)	11.3 (8.3-16.7)	<.001

Abbreviations: CNE, cumulative noise exposure; dBA, A-weighted dB; IQR, interquartile range; NA, not applicable; NG, noise-exposed group; PTA, pure-tone audiometry.

^a^ *P* values are from Kruskal-Wallis test or Pearson χ^2^ test.

### Frequency-Dependent Increase in NIHL With CNE

**Figure 2.**
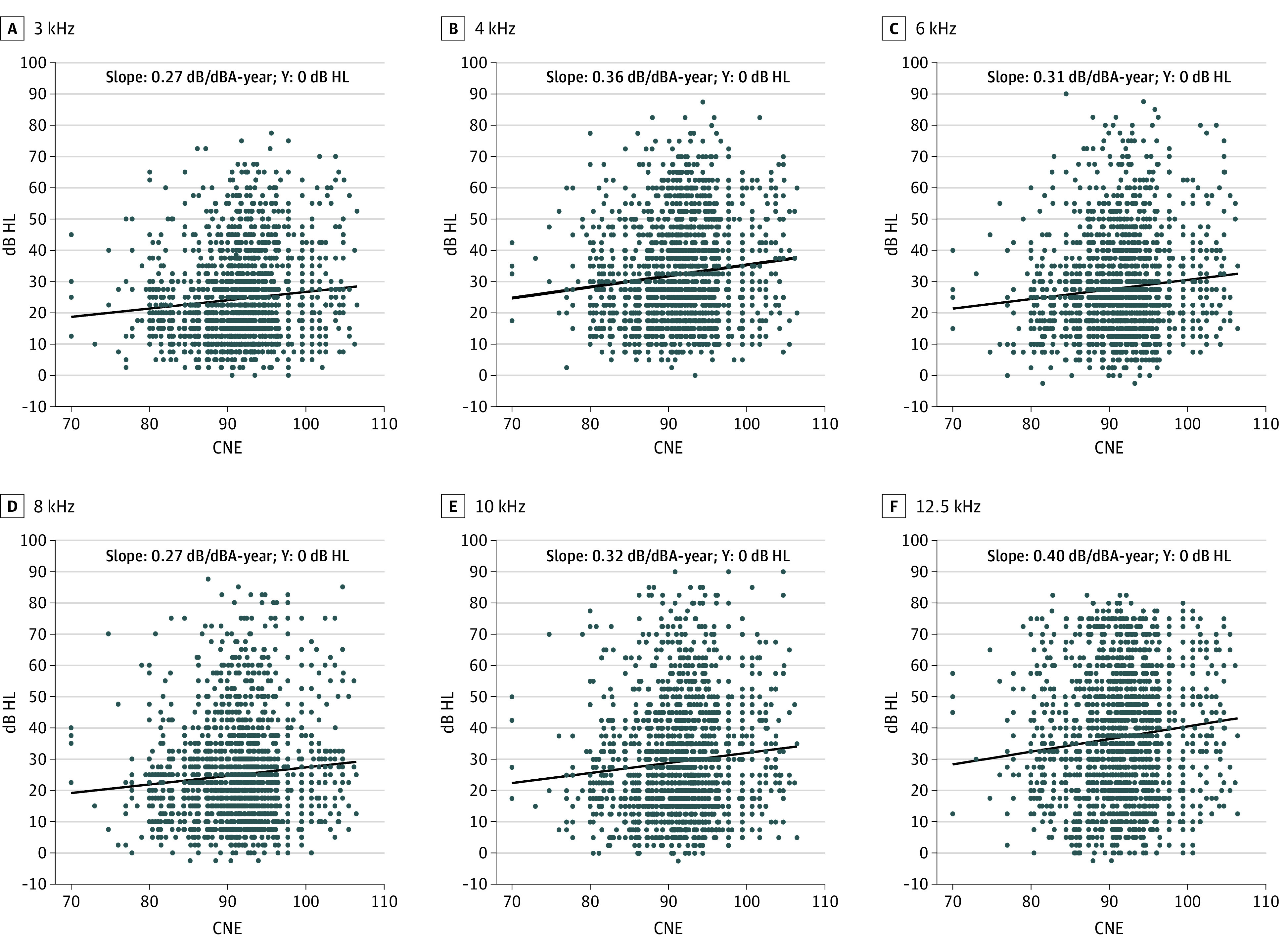
Hearing Thresholds as a Function of Cumulative Noise Exposure (CNE) at Different Frequencies Among Workers Aged 40 Years or Younger The black solid line is a fitting line with an intercept of 0. dBA indicates A-weighted dB; HL, hearing level.

To determine the association of NIHL with an individual’s CNE, which combines exposure intensity and duration, scatterplots were constructed at frequencies from 3 to 12.5 kHz showing the hearing threshold as a function of CNE for each participant. In the younger group (ie, ≤40 years), the largest slopes were 0.40 (95% CI, 0.39-0.42) dB/dBA-year at 12.5 kHz, 0.36 (95% CI, 0.35-0.36) dB/dBA-year at 4 kHz, 0.32 (95% CI, 0.31-0.33) dB/dBA-year at 10 kHz, and 0.31 (95% CI, 0.30-0.31) dB/dBA-year at 6 kHz (Figure 2). The slopes at 3 and 8 kHz were both 0.27 (95% CI for 3 kHz, 0.26-0.27; 95% CI for 8 kHz, 0.27-0.28) dB/dBA-year. The growth rate of 12.5 kHz was significantly different from the other frequencies in the younger group and the older group (12.5 kHz vs 3 kHz: *z* = 33.78; *P* < .001; 12.5 kHz vs 4 kHz: *z* = 20.39; *P* < .001; 12.5 kHz vs 6 kHz: *z* = 30.87; *P* < .001; 12.5 kHz vs 8 kHz: *z* = 32.96; *P* < .001; 12.5 kHz vs 10 kHz: *z* = 20.90; *P* < .001). The same frequency-dependent trend was present in the older group (>40 years) (eFigure 1 in the Supplement). Scatterplots were also constructed showing the hearing threshold as a function of career length and leq-8h for each participant. The same trends were found, with the maximum slope at 12.5 kHz (eFigures 2-5 in the Supplement).

### Longitudinal Progression of Hearing Loss in Younger Participants

**Figure 3.**
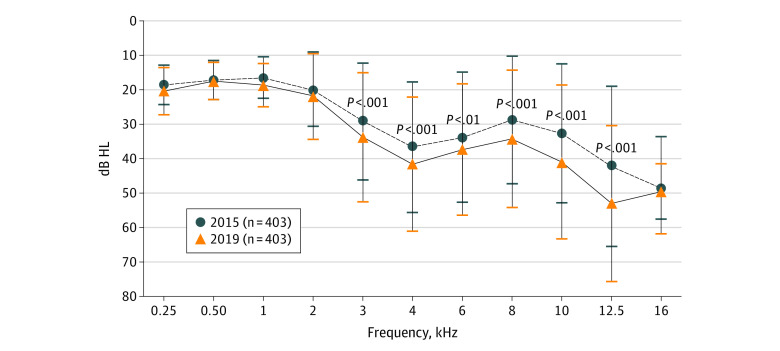
Longitudinal Progression in Hearing Thresholds of Workers Followed up From 2015 to 2019 Data are shown as means, with whiskers indicating SDs. HL indicates hearing level.

Longitudinal progression of hearing loss was plotted as a function of frequency (Figure 3). During the 4-year study period, hearing loss increased by a mean (SD) of 4.7 (8.6) dB at 3 kHz, 5.0 (8.0) dB at 4 kHz, 3.5 (8.6) dB at 6 kHz and 5.5 (9.8) dB at 8 kHz, 8.3 (12.4) dB at 10 kHz, and 10.8 (11.9) dB at 12.5 kHz. A 2-way repeated analysis of variance showed that hearing thresholds in 2019 were significantly worse than in 2015 at 3, 4, 6, 8, 10, and 12.5 kHz; the mean (SD) annual deterioration in hearing was 2.70 (2.98) dB/y at 12.5 kHz, almost twice as great as at lower frequencies (mean [SD] annual deterioration at 3 kHz: 1.18 [2.15] dB/y; 4 kHz: 1.25 [2.00] dB/y; 6 kHz: 0.88 [2.15] dB/y; 8 kHz: 1.38 [2.45] dB/y; 10 kHz: 2.08 [3.10]).

### APDs Among Workers With Noise Exposure

**Figure 4.**
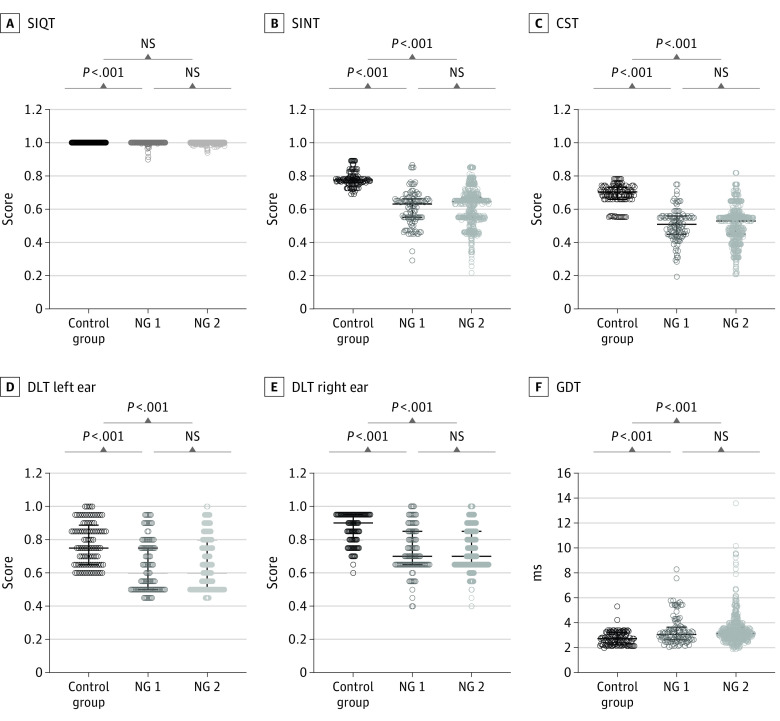
Auditory Processing Test Scores in Noise Exposed Group 1 (NG 1), Noise Exposed Group 2 (NG 2), and the Control Group CST indicates competing sentences test; DLT, dichotic listening test; GDT, gap detection threshold; NS, not significant; SINT, speech-in-noise test; SIQT, speech-in-quiet test.

The NG 1, NG 2, and control groups had similar scores on the SIQT, with no overall or between-group differences (Figure 4A). However, when the 3 groups were evaluated on the SINT (Figure 4B), the median (IQR) scores in the control group were 0.78 (0.76-0.80), whereas those in the NG 1 group were 0.63 (0.55-0.66) and those in the NG 2 group was 0.65 (0.55-0.67). The median (IQR) scores in the NG 1 group and NG 2 group were significantly less than those in the CG group (NG 1 vs control: H = 11.71; *P* < .001; NG 2 vs control: H = 14.29; *P* < .001), but there was no significant difference between the NG 1 and NG 2 groups. The median (IQR) CST score (Figure 4C) in the CG group was 0.71 (0.66-0.74), while median (IQR) scores in the NG 1 and NG 2 groups were 0.51 (0.45-0.56) and 0.53 (0.45-0.56), respectively. CST scores in the NG 1 and NG-II group were significantly less than those in the control group (NG 1 vs control: H = 11.458; *P* < .001; NG 2 vs control: H = 14.142; *P* < .001). There was no significant difference between the NG 1 and NG 2 groups. The median (IQR) score on the DL left ear was 0.75 (0.65-0.89) in the control group vs 0.60 (0.50-0.75) for the NG 1 group and 0.60 (0.50-0.80) for the NG 2 group (Figure 4D). The DL left scores for the NG 1 and NG 2 groups were significantly less than those in the control group (NG 1 vs control: H = 6.417; *P* < .001; NG 2 vs control: H = 7.754; *P* < .001), but there was no significant difference between the NG 1 and NG 2 groups. In the right ear (Figure 4E), the median (IQR) DL score was 0.90 (0.80-0.95) in the control group, whereas the median (IQR) values were 0.70 (0.65-0.85) and 0.70 (0.65-0.85) in the NG 1 and NG 2 groups, respectively. The DL right ear scores in the NG 1 group and NG 2 group were significantly less than those in the control group (NG 1 vs control: H = 9.973; *P* < .001; NG 2 vs control: (H = 7.750; *P* < .001). There was no significant difference between the NG 1 and NG 2 groups. The median (IQR) GDT values in the control group were 2.75 (2.25-3.25) ms compared with 3.08 (2.67-3.67) ms in the NG 1 group and 3.17 (2.75-3.68) ms in the NG 2 group (Figure 4F). The GDT scores were significantly longer in the NG 1 and NG 2 groups compared with those in the control group (NG 1 vs control: H = 4.891; *P* < .001; NG 2 vs control: H = 7.584; *P* < .001), but there was no significant difference between the NG 1 and NG 2 groups.

## Discussion

Among shipyard workers, we found that hearing thresholds were greater and increased more rapidly at EHFs compared with conventional audiometric frequencies. These trends were observed in both the cross-sectional and longitudinal analyses. Importantly, we found that workers with noise exposure in the NG 1 group (who had normal hearing at both clinical and EHFs) as well as workers in NG 2 (who had clinically normal hearing but elevated thresholds at EHFs) performed worse than the control group on the SINT, DLT, CST and GDT.

Hearing loss has long relied on hearing tests that evaluate sensitivity in speech frequencies. While the equipment for conducting clinical audiometric tests is widely available, that for assessing hearing at the EHFs is more expensive and not widely available. The use of EHF audiometry as an early indicator of NIHL is still controversial, but our results, as well as previous studies,^37,38^ suggest that EHF audiometry (from 9-20 kHz) could identify the first signs of NIHL, especially at 12.5 to 18 kHz. We conducted a large-scale study on shipyard workers in China aged 20 to 60 years, on the assumption that people in this age range would be old enough for noise exposure effects to be measurable but young enough that aging effects would be minimized. The univariate linear regression showed that the maximum increase of hearing loss as a function of career length, type of work setting, and CNE occurred at 12.5 kHz, not only in young workers but even in those aged of 40 years and older in the group with hearing loss.

In the longitudinal analysis (Figure 3), workers entering our longitudinal study in 2015 had the greatest hearing loss at 16 kHz; however, there was relatively little growth in hearing loss during the following 4 years, largely due to the fact that the maximum output of our transducer was 60 dB hearing level at 16 kHz so that thresholds saturated. The increase in hearing loss during the 4-year observation periods was 10.8 dB at 12.5 kHz, more than twice as that at 4 kHz (5.0 dB). These results suggest that the 12.5-kHz notch could be a more useful early indicator of NIHL than lower frequency notches.

Considerable effort has been made to identify APDs in humans with a history of noise exposure. While some studies have identified APD (eg, speech in noise, temporal processing) in individuals with a history of noise exposure,^39,40,41^ many others have reported little or no correlation between the amount of noise exposure and APD.^21,42,43,44,45,46,47^ In our study, the suprathreshold deterioration in hearing function was seen in challenging speech processing tasks such as the SINT, CST, and DLT as well as the GDT. This result was observed even in the NG 1 group, in which the PTAs in both the conventional frequency range and EHFs showed no difference from participants in the control group. One possible source of these APDs is subclinical damage that disrupts the transmission of information from the inner hair cells to the afferent auditory nerve fibers or damage to the spiral ganglion neurons, as suggested by previous human and animal studies.^26,48,49,50^ However, in the absence of histopathological data, this interpretation remains speculative.

### Limitations

This study has limitations. Some of the changes observed could be the result of aging, noise alone, or the interaction between early NIHL and aging.^51^ Further work is needed to better control for the effects of aging, but finding participants with minimal exposure to noise is becoming increasingly difficult in a world filled with numerous sound-generating devices. The definition of normal hearing in most studies is a threshold of 25 dB hearing level or less. While this may be a practical definition in a clinical setting, only those with thresholds of 0 dB (±10 dB) presumably have normal hearing. Also, there may be some bias in using self-reported data to calculate important variables.

## Conclusions

Our findings suggest that growth of hearing loss among shipyard workers was more rapid at 12.5 kHz than at 4 kHz, suggesting that 12.5 kHz could be a sensitive biomarker of early NIHL. Workers with high CNE values, even those with normal hearing at both clinical frequencies and EHFs, were more likely to exhibit APD on SINT, DLT, CST and GDT, especially when CNE values exceeded 80 dBA-year. This was observed even among workers with normal hearing in both the clinical and EHF ranges.

## Notes

Supplement. **eFigure 1.** Hearing Thresholds as a Function of CNE at Different Frequencies Among Workers Older Than 40 Years **eFigure 2.** High Frequency Hearing Thresholds as a Function of Career Length Among Workers Aged 40 Years or Younger **eFigure 3.** High Frequency Hearing Thresholds as a Function of Career Length Among Workers Older Than 40 Years **eFigure 4.** Hearing Thresholds as a Function of Career Length in the Sander Area **eFigure 5. **Hearing Thresholds as a Function of Career Length in the Welder Area
