# Supplementary material for: Analysis of Early Biomarkers Associated With Noise-Induced Hearing Loss Among Shipyard Workers
Source: JAMA Netw Open. 2021 Sep 3;4(9):e2124100. doi: 10.1001/jamanetworkopen.2021.24100 (PMC8417765; doi:10.1001/jamanetworkopen.2021.24100)
Supplement: Supplement. — eFigure 1. Hearing Thresholds as a Function of CNE at Different Frequencies Among Workers Older Than 40 Years eFigure 2. High Frequency Hearing Thresholds as a Function of Career Length Among Workers Aged 40 Years or Younger eFigure 3. High Frequency Hearing Thresholds as a Function of Career Length Among Workers Older Than 40 Years eFigure 4. Hearing Thresholds as a Function of Career Length in the Sander Area eFigure 5. Hearing Thresholds as a Function of Career Length in the Welder Area [file jamanetwopen-e2124100-s001.pdf]

# Supplemental Online Content

Jiang Z, Wang J, Feng Y, et al. Analysis of early biomarkers associated with noise-induced hearing loss among shipyard workers. *JAMA Netw Open.* 2021;4(9):e2124100. doi:10.1001/jamanetworkopen.2021.24100

**eFigure 1.** Hearing Thresholds as a Function of CNE at Different Frequencies Among Workers Older Than 40 Years

**eFigure 2.** High Frequency Hearing Thresholds as a Function of Career Length Among Workers Aged 40 Years or Younger

**eFigure 3.** High Frequency Hearing Thresholds as a Function of Career Length Among Workers Older Than 40 Years

**eFigure 4.** Hearing Thresholds as a Function of Career Length in the Sander Area

**eFigure 5.** Hearing Thresholds as a Function of Career Length in the Welder Area

This supplemental material has been provided by the authors to give readers additional information about their work.

© 2021 Jiang Z et al. *JAMA Network Open* .

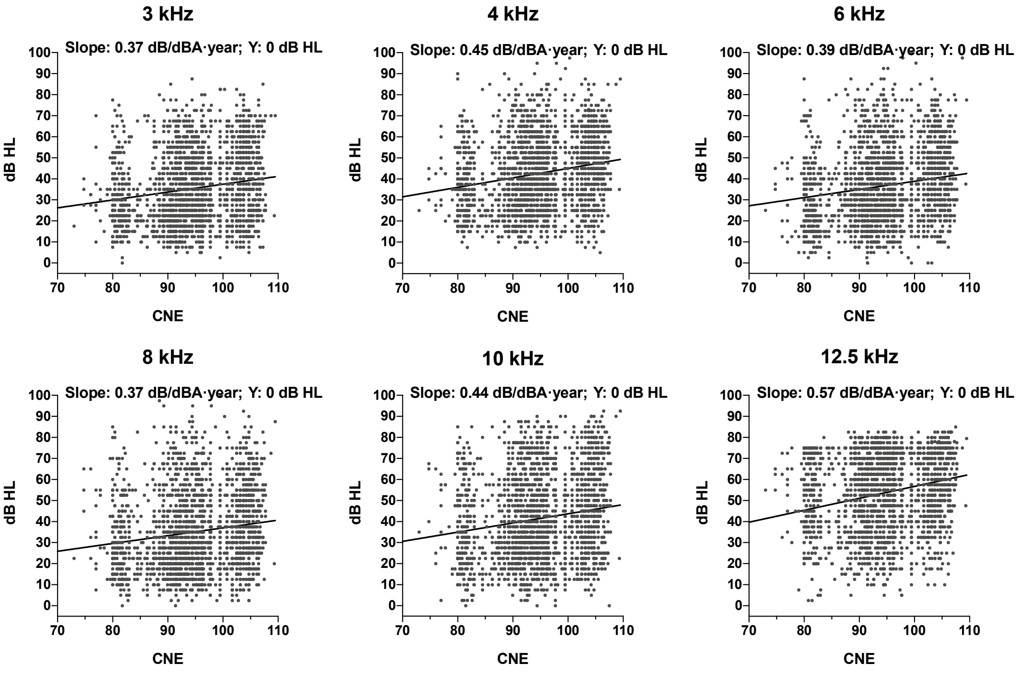


**eFigure 1.** Hearing Thresholds as a Function of CNE at Different Frequencies Among Workers Older Than 40 Years

The black solid line is a fitting line with an intercept of 0.

**Effect of career length on NIHL**

To determine how NIHL was affected by the career length, scatterplots were constructed at high frequency showing the hearing threshold as a function of career length for each subject. In the younger group (≤40 years, eFigure 2), hearing thresholds increased with career length most rapidly at 12.5 and 4 kHz, followed by 10, 6, 3 and 8 kHz; the slopes at these frequencies were 4.18 (95% CI, 4.01-4.35), 3.66 (95% CI, 3.53-3.80), 3.30 (95% CI, 3.15-3.44), 3.07 (95% CI, 2.94-3.20), 2.78 (95% CI, 2.67-2.89), and 2.78 (95% CI, 2.65-2.90) dB/year. In the older group (>40 years, eFigure 3), hearing thresholds also increased with career length most rapidly at 12.5 and 4 kHz, followed by 10, 6, 3 and 8 kHz; the slopes at these frequencies were 4.50 (95% CI, 4.38-4.62), 3.60 (95% CI, 3.50-3.70), 3.50 (95% CI, 3.39-3.60), 3.04 (95% CI, 2.95-3.14), 3.00 (95% CI, 2.91-3.09), and 2.95 (95% CI, 2.85-3.04) dB/year.

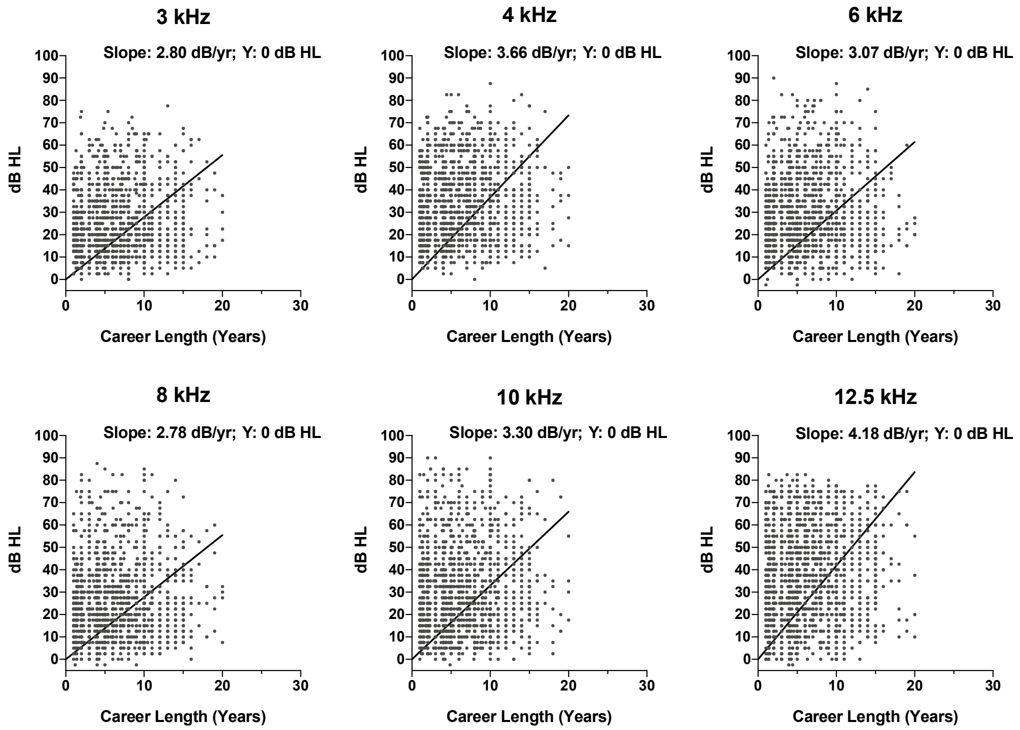


**eFigure 2.** High Frequency Hearing Thresholds as a Function of Career Length Among Workers Aged 40 Years or Younger

The black solid line is a fitting line with an intercept of 0.

© 2021 Jiang Z et al. *JAMA Network Open* .

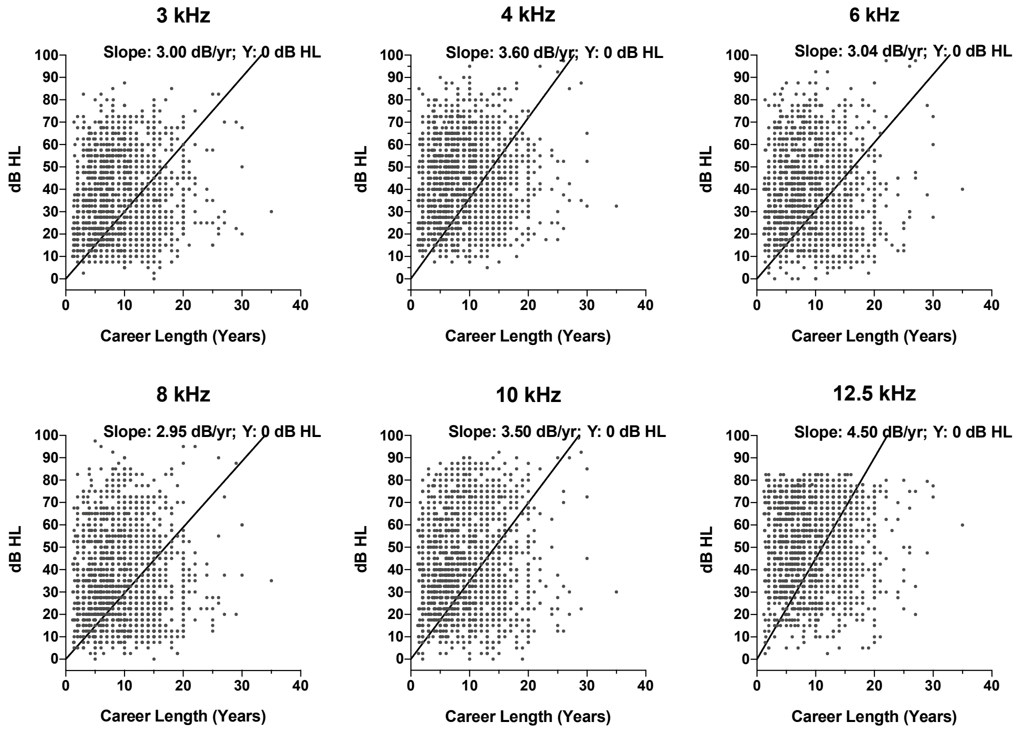


**eFigure 3.** High Frequency Hearing Thresholds as a Function of Career Length Among Workers Older Than 40 Years

The black solid line is a fitting line with an intercept of 0.

**Effect of occupational Leq-8h level on NIHL**

Because the magnitude of NIHL has long been known to be strongly dependent on exposure intensity, scatterplots were constructed to show the effect of occupational Leq-8h level on NIHL for the age-matched young workers in the higher noise Sander area (~95 dBA, n=288) and the lower noise Welder area (~82 dBA, n=547). There was no significant difference in age (mean [SD]) between the Sander (32.5 [4.6] years) and Welder workers (32.4 [4.8] years) ($p>0.05$). Among the Sanders (eFigure 4), the slopes showing the growth rate of hearing loss with career length was 4.80 (95% CI, 4.33-5.27), 5.98 (95% CI, 5.42-6.54), 5.48 (95% CI, 4.97-5.99), 4.90 (95% CI, 4.39-5.44) 5.68 (95% CI, 5.09-6.26) and 6.70 (95% CI, 6.02-7.38) dB/year for 3, 4, 6, 8, 10 and 12.5 kHz, respectively. For the Welders (eFigure 5), the slopes were 2.53 (95% CI, 2.36-2.70), 3.54 (95% CI, 3.33-3.76), 3.00 (95% CI, 2.79-3.21), 2.78 (95% CI, 2.58-

2.98), 3.32 (95% CI, 3.08-3.55) and 4.37 (95% CI, 4.08-4.65) dB/year for 3, 4, 6, 8, 10 and 12.5 kHz, respectively. In general, the slopes were steeper for workers in the high-noise-level Sander area than the low-noise-level Welder area. Moreover, the slopes were steeper in both Sander and Welder areas at 12.5 kHz, and 4 kHz.

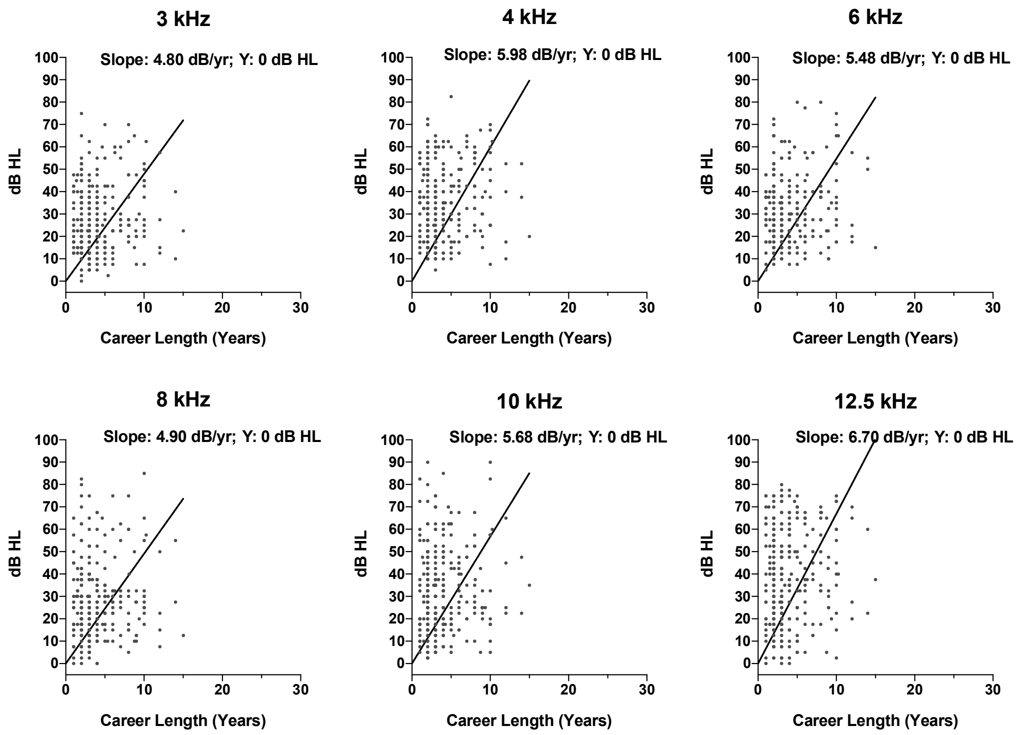


**eFigure 4.** Hearing Thresholds as a Function of Career Length in the Sander Area. The black solid line is a fitting line with an intercept of 0.

© 2021 Jiang Z et al. *JAMA Network Open* .

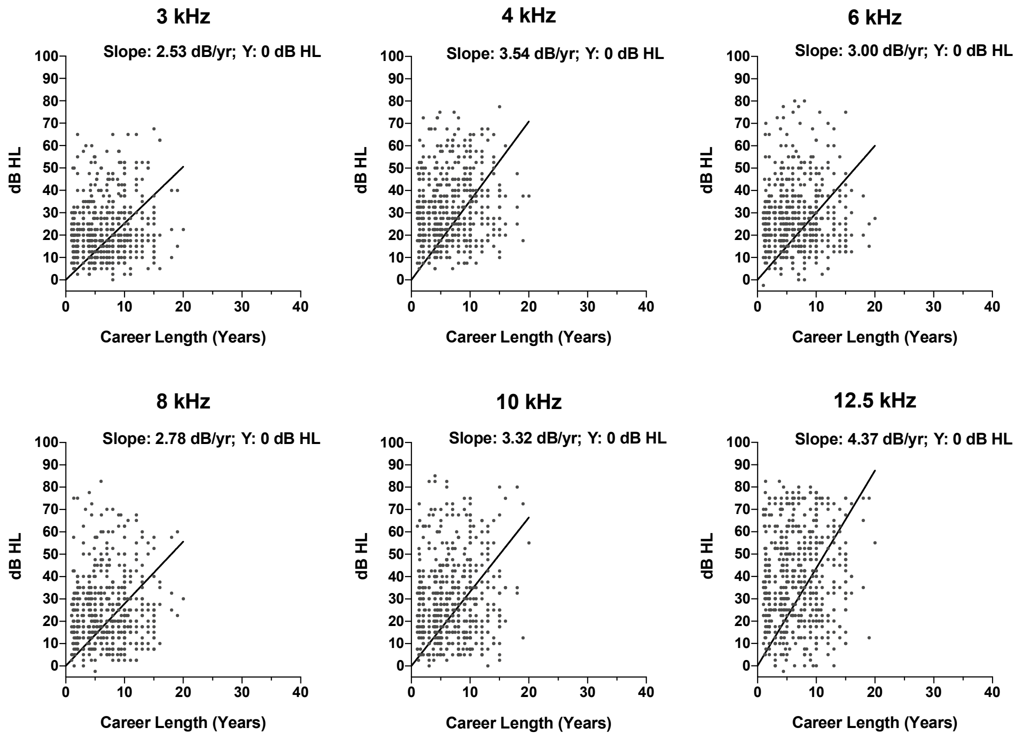


**eFigure 5.** Hearing Thresholds as a Function of Career Length in the Welder Area

The black solid line is a fitting line with an intercept of 0.

© 2021 Jiang Z et al. *JAMA Network Open* .
